# Serosal overturning assisted endoscopic full‐thickness mucosal resection of extraneous giant mass at the esophagogastric junction

**DOI:** 10.1002/ccr3.9226

**Published:** 2024-08-06

**Authors:** Shumin Qin, Xiaofeng Lin, Shuting Wen, Tianwen Liu

**Affiliations:** ^1^ Department of Gastroenterology The Second Affiliated Hospital of Guanzhou University of Chinese Medicine Guangzhou China

**Keywords:** endoscopic full‐thickness mucosal resection, Esophagogastric junction, extraneous giant mass, Serosal overturning

## Abstract

**Key Clinical Message:**

Serosal overturning assisted endoscopic full‐thickness mucosal resection was performed on the extraneous giant masses at the esophagogastric junction without complications.

**Abstract:**

It is difficult to perform endoscopic resection of masses at the gastroesophageal junction (GEJ). In particular, the extraneous giant masses surrounding the extraneous giant masses is infrequent. As one of the technologies of endoscopic resection, endoscopic full‐thickness resection (EFTR) is generally applicable to the submucosal tumor of stomach, duodenum and colorectal that originate from the musculus propria and protrude to subserous or partial growth outside the luminal layer. Successful endoscopic repair of perforation is crucial in avoiding the need for surgical repair and preventing postoperative peritonitis, making it a key aspect of EFTR treatment. We report a 56‐year‐old woman who was admitted to our department complaining of 5‐year history of masses of esophagogastric junction and 2‐month history of feeling of gastric distension. Gastroscopy showed a 4 cm submucosal mass near the fundus of the stomach from the cardia. Computed tomography scan revealed submucosal lesions in esophagogastric junction, which was exogenous. We successfully performed Serosal overturning assisted endoscopic full‐thickness mucosal resection on the extraneous giant masses at the esophagogastric junction without complications. The clinical symptoms were significantly improved within postoperative 1 month. There was no recurrence 8 months after the operation. Serosal overturning assisted EFTR is possibly an effective and minimally invasive method of extraneous giant masses at the esophagogastric junction.

## 1 INTRODUCTION

Due to the specific anatomical structure of the esophagogastric junction, it is regarded as one of the most difficult parts in endoscopy examination.^1^ Stripping the mass at the esophagogastric junction requires more accurate and stable endoscopic operation. Furthermore, there is a high risk of an exogenous mass falling into the abdominal cavity. The larger the tumor, the longer and more difficult the operation of endoscopic resection, and the higher the risks of intraoperative perforation, postoperative infection and delayed bleeding.^2^ For submucosal tumor at the esophagogastric junction, endoscopic full‐thickness resection (EFTR) can treat deeper lesions of the digestive tract and expand the scope of endoscopy treatment. We encountered a woman with a giant extraneous mass at the esophagogastric junction. The mass was successfully excised by EFTR assisted with serosal overturning, without postoperative complications.

## 2 CASE HISTORY/EXAMINATION

**FIGURE 1:**
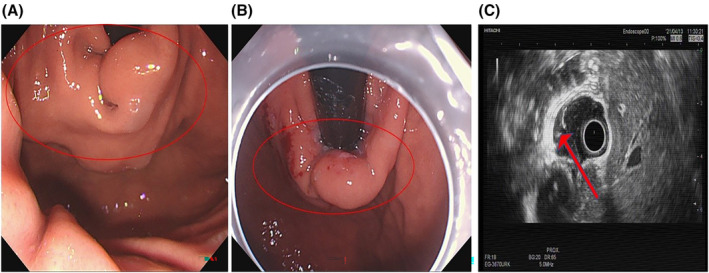
Endoscopy. (A) An endoscopic view from 2016: Protruding mass with a smooth surface was observed in the esophagogastric junction. (B) An endoscopic view from 2021: Protruding mass with slightly hyperemic surface was observed in the esophagogastric junction. (C) Endoscopic ultrasonography from 2021: A mass was derived from the musculus propria and surrounded about half of the cavity, with irregular shape.

**FIGURE 2:**
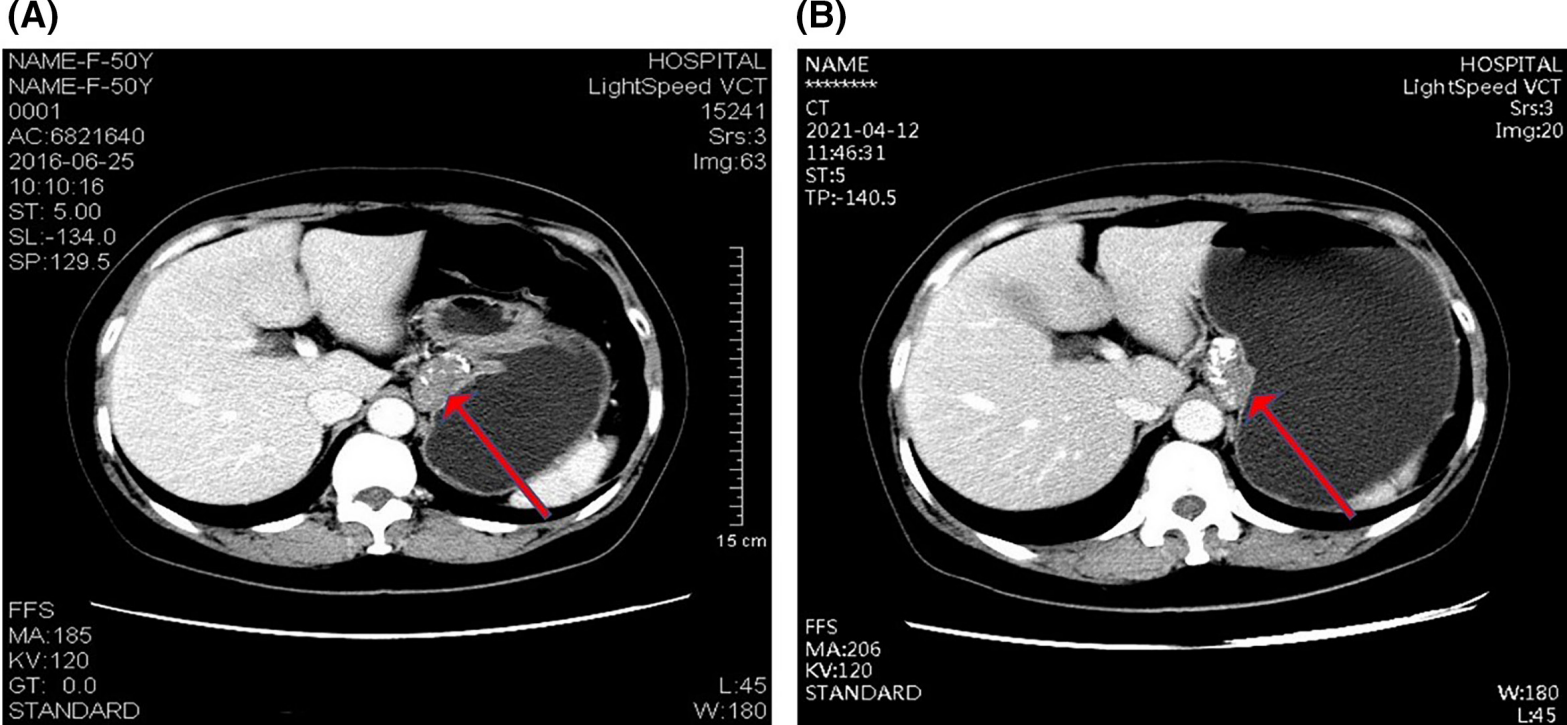
Conventional and enhanced computed tomography (CT) images. A submucosal mass protruding into the abdominal cavity was seen in esophagogastric junction. (A) the CT image 5 years ago; (B) the CT image on admission.

A 56‐year‐old woman admitted to the Department of Gastroenterology of The second affiliated hospital of Guangzhou University of Chinese Medicine. She had 5‐year history of mass at the esophagogastric junction and 2‐month history of feeling of gastric distension. Five years ago, she was found to have a mass at the esophagogastric junction by endoscopy and contrast‐enhanced thoracic computed tomography (CT) (Figure 1A and Figure 2A). However, she did not take any treatment because negligence without any symptom. Gastroscopy on admission showed a 4‐cm submucosal mass near the fundus of the stomach from the cardia (Figure 1B). The surface was smooth and the contact was hard and immobile. As shown in Table 1, physical examination and laboratory findings were unremarkable. Contrast‐enhanced whole abdomen CT scan revealed a submucosal lesion at the esophagogastric junction (Figure 2B), which was slight larger compared with the images 5 years ago (3.6 cm × 2.2 cm vs. 3.5 cm × 2.2 cm). There was a low‐density fat gap with nonuniformity of image density and multiple nodular density shadow between pancreatic neck and the mass (Figure 2). Endoscopic ultrasonography (EUS) demonstrated that the mass was derived from the musculus propria and surrounded about half of the cavity, with irregular shape. About half of the lesions were located outside the cavity with inhomogeneous low‐echo light group, and multiple patchy hyperecho were observed outside the cavity (Figure 1C).

**TABLE 1:** Laboratory observations upon admission.

Characteristics	Index	Reference range
Blood		
WBC count (_10^9^/L)	7.18	3.50–9.50
RBC count (_10^12^/L)	3,91	3.80–5.10
Hb (g/L)	121	115–150
PLT count (_109/L)	233	125–350
Coagulation function		
PT (s)	10.9	10.0–13.0
APTT (s)	24.9	24.0–32.8
Liver function		
ALT (m/L)	8	7–40
AST (m/L)	11	13–35
G‐GT (m/L)	13	7–45
ALP (m/L)	62	50–135
ALB (g/L)	45.9	40.0–55.0
Serum tumor markers		
AFP (ng/mL)	5.2	0.00–7.00
CEA (ng/mL)	1.61	0–5
CA125 (U/mL)	13.66	0.00–25.00
CA153 (U/mL)	3.64	0.00–24.00
CA199 (U/mL)	4.89	0.00–30.00

Abbreviations: AFP, alpha fetoprotein; ALB, albumin; ALP, alkaline phosphatase; ALT, alanine transaminase; APTT, activated partial thromboplastin time; AST, aspartate aminotransferase; CA125, carbohydrate antigen 125; CA153, carbohydrate antigen 153; CA199, carbohydrate antigen 199; CEA, carcinoembryonic antigen; G‐GT, glutamyl transpeptidase; Hb, hemoglobin; PLT, platelet; PT, prothrombin time; RBC, red blood cell; WBC, white blood cell.

## 3 METHODS

After obtaining informed consent, EFTR combined with lower serosal overturning was carried out by aspiration lumpectomy.

## 4 CONCLUSION AND RESULTS

The resected specimen was a semicircular strip‐type of mass with the volume of 16 × 3 cm. Immunohistochemistry staining revealed DOG1 (−), SMA (+), Desmin (+), CD34 (partially+), CD117(−), S‐100(−), Ki67(<1%+). The clinical symptoms improved significantly within postoperative 1 month. Repeated endoscopy at 6 months showed normal in the stomach and esophagus.

## 5 DISCUSSION

The gastroesophageal junction (GEJ), a 2‐cm interface between the distal esophagus and the gastric fundus, predominantly features a muscularis propria layer. The majority of esophageal submucosal tumors originating in this region are benign; however, a minority exhibit malignant potential.^3^ Current clinical guidelines widely recommend early resection of muscularis propria tumors at the GEJ.^4^ Laparoscopy can effectively treat tumors that grow into the abdominal cavity.^5^ However, it must remove part of the esophagus, resulting in the destruction of the inherent anatomy and functional structure of the GEJ.^6^, ^7^ Open surgical procedures could cause more trauma to the body and a higher incidence of complications.^8^, ^9^ Endoscopic resection of muscularis propria masses in the digestive tract offers an advantage of preserving the cardia and esophagus, while minimizing tissue damage, promoting rapid recovery, and generally yielding favorable outcomes.^10^, ^11^, ^12^

The current endoscopic treatments for propria tumors include endoscopic submucosal excavation (ESE), EFTR, and submucosal tunnel endoscopic resection (STER). ESE, an evolution of endoscopic submucosal dissection (ESD), is predominantly indicated for submucosal tumors with a diameter of 2 cm or greater.^13^ Studies have shown a high complete resection rate, exceeding 90%, for ESE in submucosal tumors,^14^, ^15^, ^16^ with perforation being the most common complication.^16^, ^17^ STER, built upon peroral endoscopic myotomy (POEM) technology, represents an extension of ESD techniques, particularly for esophageal and gastric submucosal tumors originating from the muscularis propria with diameters below 5 cm. The reported overall resection rate for STER in these tumors ranges from 78% to 100%, with gas‐related complications and pleural effusion being the primary concerns.^18^, ^19^, ^20^ Independent risk factors for incomplete resection include irregular tumor morphology, deep muscularis propria origin, intraoperative air perfusion, and procedures lasting more than 60 min.^18^ EFTR is typically employed for submucosal tumors in the stomach, duodenum, and colorectal regions, originating from the muscularis propria. CT scans indicate tumors extending to the subserosal layer or partially protruding beyond the luminal surface. EUS reveals tight adherence to the serosal layer, making separation challenging, Moreover, EFTR is indicated for esophageal submucosal tumors with diameters exceeding 5 cm, where STER is not applicable. The reported complete resection rate for EFTR in these tumors ranges from 87.5% to 100%, with a low incidence of complications, including a few reported cases of abdominal infection postprocedure.^21^, ^22^

The current case study documents an atypical esophagogastric junction mass. Both the CT scan and EUS findings revealed the tumor's extension beyond the serosal margin and partial infiltration into the submucosal layer, indicating its eligibility for EFTR. Hence, we opted for EFTR as the treatment modality. Success in EFTR is predicated on effectively managing perforation, preventing surgical intervention, and minimizing postoperative peritonitis, as highlighted in studies.^2^, ^23^ At present, the metal clip stitching, aspiration‐clipping suture, omental patch suture, and string suture are the main suture techniques for EFTR.^23^ The metal clip is the most basic suture tool in EFTR repair, applied to completely suture the wound from both sides to the center under endoscopic direct vision. Due to the limited span of the metal clip, multiple metal clips are used to close the perforation, also known as “aspiration‐clipped suture.”^24^ If the wound is too large, negative pressure can be applied to attract the omental momentum into the digestive tract cavity. Metal clips can then be used to clamp the omental patch and mucosa along the edge of the wound to close it, a procedure known as “omental patch suture.” The string suture is performed under double forceps endoscopy. In one forceps passage, nylon rope is placed around the incisor edge of the gastric wall. In the other forceps passage, multiple metal clips are placed to close the mucosal tissue and secure the nylon rope at the incisor edge. Finally, the nylon rope is tightened to close the wound.^25^

**FIGURE 3:**
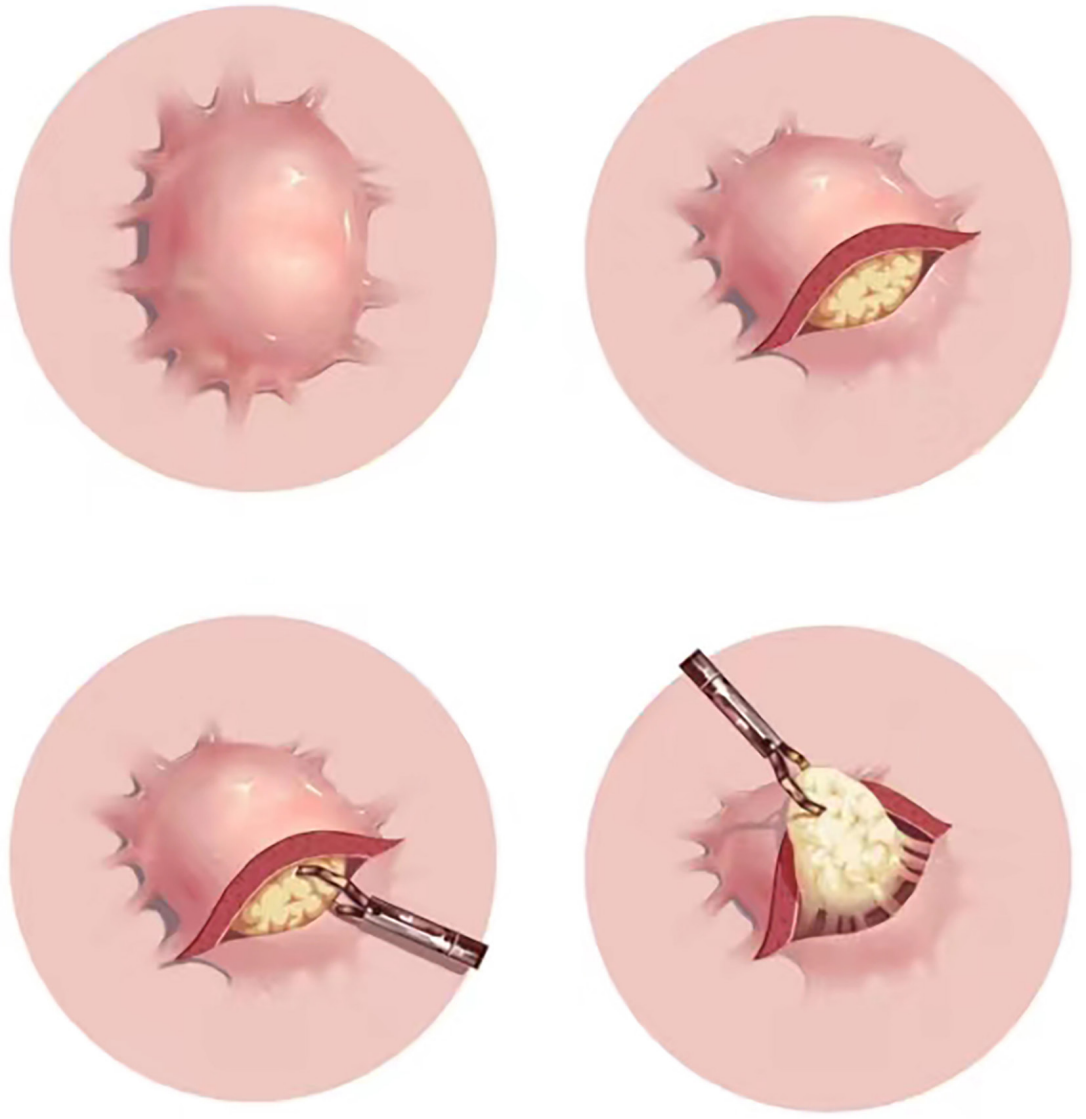
Concise sketch map of the “serosal overturning.”

In the present case, the metal clip stitching and aspiration‐clipping suture were applied. Additionally, a new method named “serosal overturning” was used to assist with the suturing. When serosal protrusions are outside the lumen, they are abundant in blood vessels. It is challenging to deal with the exposed blood vessels of the serosa since the endoscope in the cavity cannot directly view the serous membrane outside the cavity at the perforation. “Serosal overturning” involves fixing the serosa at the lower end of the perforation with a metal clip at one end, pulling the clip and serosa into the cavity with thread at the other end, and then solidifying and clamping the exposed blood vessels with metal clips (Figure 3). This method helps reduce bleeding during surgery and shorten the time for suturing the perforation.

In conclusion, we described an unusual presentation of an extraneous giant mass at the esophagogastric junction. In addition, a new method named “serosal overturning” was used to assist with suturing. The Serosal overturning assisted EFTR is possibly an effective and minimally invasive method of extraneous giant mass at the esophagogastric junction.

## AUTHOR CONTRIBUTIONS

**Shumin Qin:** Data curation; methodology; writing – original draft; writing – review and editing. **Xiaofeng Lin:** Data curation; methodology. **Shuting Wen:** Formal analysis; methodology. **Tianwen Liu:** Conceptualization; methodology; supervision.

## FUNDING INFORMATION

None.

## CONFLICT OF INTEREST STATEMENT

The Authors declare that there is no conflict of interest.

## ETHICS STATEMENT

This work does not involve any human/animal experimentation.

## CONSENT

We've obtained a signed informed consent from the patient according to the journal's patient consent policy.

## Data Availability

The data that support the findings of this study are delivered by the corresponding author, upon reasonable request.
